# Barriers to sexual health care: a survey of Iranian-American physicians in California, USA

**DOI:** 10.1186/s12913-016-1481-8

**Published:** 2016-07-15

**Authors:** Mitra Rashidian, Victor Minichiello, Synnove F. Knutsen, Mark Ghamsary

**Affiliations:** Collaborative Research Network, University of New England, School of Health, Armidale, Australia; School of Medicine and Public Health, University of Newcastle, Newcastle, Australia; University of New England, Armidale, Australia; Department of Biostatistics and Epidemiology, Loma Linda University, School of Public Health, Loma Linda, USA

**Keywords:** Iranian-American physicians, Access to care, Sexual health care, STIs, Sexual dysfunctions

## Abstract

**Background:**

Despite increasing numbers of Iranian-American physicians practicing in the United States, little is known about the barriers that may impact them as providers of sexual health care. This is an important topic as discussions of sexual topics are generally considered a taboo among Iranians. We aimed to identify barriers experienced by Iranian-American physicians that inhibit their willingness to engage in discussions of sexual health care with patients.

**Methods:**

In 2013, a self-administrated questionnaire was sent to 1,550 Iranian-American physicians in California. Questions included demographics of the physicians as well as their perception of challenges in discussing various sexual health topics with their patients. Factor analysis: Principal components approach with a Varimax rotation was used to detect latent factors within the data that may help explain possible barriers to discussion of sexual health among physicians. The analysis was performed on 11 items, specifically focused on possible barriers, to detect a possible relationship between correlated variables within the data to produce a set of uncorrelated variables (factors).

**Results:**

The overall response rate was 23 %. Data revealed specific barriers regarding sexual history taking, discussing STIs and sexual dysfunctions with patients based on their gender, and age. Three factors were identified as internally consistent (Cronbach’s alpha = 0.82 to 0.91): (i) embarrassment, (ii) cultural and religious, (iii) lack of time and financial constraint. Significant associations were found between these 3 factors and some variables such as: country of medical graduation, religious affiliation, birthplace, age, and gender.

**Conclusions:**

Our findings are the first to identify possible barriers among Iranian-American physicians in delivering effective sexual health care to patients. Additional studies from Iranian-American physicians as well as from other foreign-born/subpopulation of US physicians populations and mainstream US physicians are needed to assess the extent of such barriers, and changes over time. Effective strategies to better engage such physicians in these studies are needed. If confirmed from other studies, our findings could have implications for the training of US medical graduates.

**Electronic supplementary material:**

The online version of this article (doi:10.1186/s12913-016-1481-8) contains supplementary material, which is available to authorized users.

## Background

Sexual health and quality of life are directly related [1]. Sexual history taking as part of holistic care, however, is not routinely addressed in health care services [2]. According to the Centers for Disease Control and Prevention (CDC) [3, 4], 20 million new sexually transmitted infections occur in the United States each year and in 2013 the total number of STIs among US men and women exceeded 110 million. Among those 15 years and older, these estimates included eight common STIs: chlamydia, gonorrhoea, hepatitis B virus (HBV), herpes simplex virus type 2 (HSV-2), human immunodeficiency virus (HIV), human papillomavirus (HPV), syphilis, and trichomoniasis, with 49 % of incident STIs occurring among young men, vs. 51 % among young women [3, 5]. Research suggests that the lack of a unified approach to sexual health is indicative of a poor sexual health care system within the United States [6]. As a result, the rates of STIs and sexual dysfunctions are higher compared to many other developed countries [7, 8]. Of the nearly 20 million new STIs every year in this country, half occur among young people ages 15–24. In addition to increasing a person’s risk for HIV infection, STDs can lead to severe reproductive health complications, such as infertility [9–11]. Research further suggests that where HIV prevalence is high, rates of unintended pregnancy and unsafe abortion are also high resulting in an increase in maternal deaths as well as long-term health consequences [9, 10]. Physician-patient communication is of fundamental importance in health care [11, 12]. Increased communication correlates with increased use of condoms, whereas lack of communication is a risk factor for HIV and STIs [12, 13].

Failure to correctly diagnose patients with sexual health problems is often due to barriers among medical professionals such as dealing with follow-up, costs and how to do appropriate testing [14]. One study suggests that physicians frequently underestimate the pervasiveness of sexual concerns among their patients [1] and only about 6 % of physicians initiate discussion on this topic on a regular basis [15]. Among US physicians, there is evidence that physicians such as general practitioners do not address sexual health issues proactively with patients [12]. Studies show that patients would like to discuss sexually-related issues with their physicians, but are often reluctant because they fear that the physician will be embarrassed, or will dismiss their concerns [12, 16].

The absence of proactive communication is believed to be due to a lack of training in effective sexual health care [17]. Known factors that serve as barriers to sexual health care include: 1) health care organizational factors such as complexity, time constraints, training and expertise, 2) structural factors such as economics and politics, and 3) the sensitivity of the topic and its impact on the health care practitioners’ personal motivation which could impede or facilitate discussion with patients [2]. The identified barriers also included reluctance in discussing sexual health care with minority groups.

Aside from proactive communication skills and the need for increased education about various aspects of sexual health such as sexual dysfunctions and STIs [18], some of the other identified barriers among US physicians include: underestimation of patient risk; inadequate and/or insufficient knowledge of sexual health; and lack of privacy [19]. Conservative sexual beliefs and cultural biases [20], the gender of both the physicians and the patients, fear of intrusion [5, 21, 22], and age [23] are additional barriers identified among US physicians. More than one-third of new HIV infections occur among those aged 13-29 years as they continue to engage in risky sexual behaviours [24, 25], and 1 in 4 sexually active adolescent females have an STD such as chlamydia or human papillomavirus (HPV) [26]. Compared with older adults, sexually active adolescents aged 15–19 years and young adults aged 20–24 years are at higher risk of acquiring STDs due to a combination of behavioural, biological, and cultural factors. While various forms of barriers to proactive communication are known as lack of cultural competency among the mainstream US physicians [27], literature is limited on US physicians from minority backgrounds with respect to the impact of their cultural backgrounds on the delivery of sexual health care within the USA. A study at the University of California Los Angeles (UCLA) indicated that white primary care providers, were less likely than Hispanic/Asian/African- American/other, to regularly take sexual histories from their patients. Perceived key barriers among physicians included patient’s young age (<16 years), language, and the presence of patient’s relative/partner in the consultation room at time of visit [28]. The results of a related study among primary care physicians in four specialties (obstetrics/gynecology, internal medicine, general/family practice, pediatrics), plus urogynecologists [29], showed that these health providers often refrain from doing sexual histories as part of routine and preventive healthcare because they feel uncomfortable. Thus, many physicians miss essential components of a comprehensive sexual history [29, 30].

Having physicians of various foreign-born/subpopulation minority backgrounds is believed to be positive in providing effective sexual health care as research shows that patients who are members of minority groups are more likely than others to consult physicians of the same race or ethnic group [31]. Research indicates that among the US minority physicians, Black and Hispanic physicians have a unique and important role in caring for poor Black and Hispanic patients in California [31, 32]. Foreign-born/minority background physicians may have additional barriers such as personal factors that are reinforced by cultural biases and personal beliefs. These again may be reinforced by a larger societal view, plus personal upbringing and religious beliefs that physicians may hold about sexuality, and therefore about sexual health care [33–35]. Therefore, exploring how foreign-born physicians connect to their patients in the area of sexual health care can uncover helpful strategies that all physicians and those of diverse background can use to establish stronger cross-cultural connections and communication levels. Physicians’ gender also has been considered as a barrier of care when patients are of the opposite gender, very young, or older [36].

The Iranian-American physicians are among the foreign-born/minority medical doctors within the US. Whether they hold similar value system and biases on sexuality, impacting the delivery of sexual health care is relatively unknown. Research suggests that the discussion of sexuality is taboo within the Iranian culture [37–39] and therefore it is important to survey Iranian-American physicians to assess to what degree they experience barriers on this topic in their daily interaction with patients. The communication with patients concerning sexual care in Iran is primarily limited to reproductive care among patients and their physicians [37]. This narrow practice of sexual health care could potentially serve as a barrier to having a proactive communication system for Iranian-American physicians when discussing patients’ sexually-related concerns, in particular with the opposite gender.

Iranian-Americans are Americans of Iranian ancestry and/or people holding Iranian and American dual citizenships. In 1971, Iranian physicians in the U.S. numbered 1,625 [40]. Later the majority of the Iranian-American physicians migrated to the United States subsequent to Iran’s revolution in 1979. Those who immigrated to the US were generally qualified physicians who came with their families with the intent to stay permanently. Of these, at least 5,000 received their primary medical degree in Iran, with advanced training in the United States after immigration [41]. According to the 2010 US Census, it is estimated that there are at least 10,000 Iranian-American physicians across the United States with the vast majority currently practicing in California. The number of Iranian Medical School graduates in the United States had grown to 5,045 post-revolution [42], and they practice primarily in the areas in which most of the Iranian American population is concentrated [40]. Aside from practicing physicians, Iranian immigration to the United States has been continuous since the 1980s. Between 1980 and 1990, the number of Iranian-born people in the United States increased by 74 % [43]. Currently, the United States contains the highest number of Iranians outside of Iran. Iranian-Americans regard their culture and heritage as an important component of their daily life and of their overall identity within the United States [44].

The existing literature on the delivery of sexual health care given by Iranian physicians in Iran is known to be limited due to various barriers such as social stigmatization of sexually related topics [45]. Some studies [46–48] proposed that proactive communication about sexually related issues by Iranian physicians plays a vital part in creating rapport with patients. Limited communication about sexuality has led to a lack of research on the topic and a culturally state-imposed position on sexuality frequently adopted by Iranian physicians in Iran. When coupled with homophobia and sexism, this may subject patients to potential sexual health risks [37, 48, 49]. Many of Iranian-Americans may seek medical help from the Iranian-American physicians due to the English language limitations. The Iranian-American adolescents may have conflicting views with their parents about the nature of sexual health care. In particular, while families and the Iranian-American communities continuously put emphasis on retaining Iranian traditional values regarding sexual issues, through direct exposure to host culture, the Iranian-American adolescents have identified with the sexual values introduced to them in the United States [50].

To date, no data has been reported about possible barriers that may prevent Iranian-American physicians from discussing sexually related concerns with their patients within the US. Our pilot study, to the best of our knowledge, is the very first one to focus on this issue. Research about the nature of sexual health care offered by Iranian-American physicians to Iranian-American patients as well as to the patients of mainstream and diverse ethnic backgrounds, is also unknown. To assess and to identify possible barriers, we conducted a survey of Iranian-American physicians in California, where the majority of these physicians have their practices, to learn more about how they relate to their role as providers of sexual health care within the United States. We acknowledge that this is a difficult and sensitive topic for this population and thus has implications for response rates. However, there is an urgent need to conduct such studies in order to better understand and address possible barriers and thus improve sexual health care delivery among this group of health care providers. Focusing on impediments to appropriate sexual health care will also make it easier to conduct larger studies among both Iranian-Americans and other subgroups of US physicians.

## Methods

### Study design

This is a descriptive cross-sectional study of Iranian-American physicians practicing in California. A structured self-administered questionnaire was designed to collect information related to factors that may act as barriers to the provision of sexual health care among Iranian-American physicians. Barriers to sexual health care is defined as anything standing in the way of providing sexual health care.

### Study population

The target study population was Iranian-American physicians (a) who, based on their specialty (family practice, internal medicine, obstetrics/gynaecology, paediatrics and dermatology/venereology, endocrinology, geriatrics, plastic surgery, proctology, psychiatrics, psychology, and urology), would potentially offer sexual health care to their patients, (b) who are currently practicing in California, and (c) who available and willing to participate. The process of identifying potential participants included collecting names and addresses across California using public information. The current Iranian registry lists available via the Internet, phone, published physical address, and email include: Anthem Blue Cross of California Insurance Company, Beverly Hills Iranian-American Doctors (BHIAD), Iranian-American Doctors Directory, Iranian-American Medical Association (IAMA), Academy of Persian Physicians, and the Iranian American Society. Other resources included the US yellow pages, the Iranian yellow pages published annually by the Ketab Corporation in Los Angeles, and assistance from the University of California Irvine (UCI), School of Medicine, and Loma Linda University, School of Public Health and School of Medicine. Through these mechanisms, a total of 1,550, of approximate 5,000 Iranian-American physicians in California, met the inclusion criteria and these were the target for this study. Since there is no formal power analysis for factor analysis [51], we attempted to survey the full sample of 1,550 [52, 53] health care practitioners (see Table 1).

**Table 1:** Physician characteristics

	*n* (%)
Gender	
Male	203 (57.3 %)
Female	132 (37.3 %)
Missing	19 (5.4 %)
Age	
30 – 39 years	36 (10.2 %)
40 – 49 years	110 (31.1 %)
50 – 59 years	71 (20.1 %)
60 – 69 years	97 (27.4 %)
70 – 89 years	15 (4.2 %)
Missing	24 (7.1 %)
Place of Birth	
Iran	291 (82.2 %)
Other	35 (9.9 %)
Missing	28 (7.9 %)
Country of Medical Education	
Iran	190 (53.8 %)
USA	130 (36.8 %)
Other	8 (2.3 %)
Missing	25 (7.1 %)
Location of Medical Practice	
Suburban	175 (49.6 %)
Urban	138 (39.1 %)
Rural	20 (5.7 %)
Missing	20 (5.7 %)
Religion of Physician	
Muslim	192 (54.4 %)
Jewish	77 (21.8 %)
Other	64 (18.1 %)
Missing	20 (5.7 %)
Clinical Specialty of Physician	
Family practitioner	62 (17.5 %)
Internist/cardiologists	56 (15.8 %)
Obstetrician/gynaecologist	53 (15 %)
Pediatrician (youth care)	30 (8.5 %)
Urologist	30 (8.5 %)
Dermatologist/venerologist	23 (6.5 %)
Gastrologist	18 (5.1 %)
Psychiatrist	18 (5.1 %)
Other	15 (4.2 %)
Geriatrics	11 (3.1 %)
Plastic surgeon	11 (3.1 %)
Missing	18 (7.6 %)

### Questionnaire development

This paper is part of a larger study on sexual health care among this population. A questionnaire was developed specifically for our study in accordance with standardized surveys on sexual health care designed by the Center for AIDS Prevention Studies (CAPS), Survey Instruments and Scales for providers [54]. Based on a review of sexual health care literature, four themes were used for the instrument: 1) Sexual Health Care –This section consists of the following sub-sections: a) Obtaining Sexual History; b) Physicians’ Attitudes; and c) Education/Counseling; d) Sexually Transmitted Infections (STIs). The 2^nd^ theme was Sexual Health Training which included four questions related to the delivery of sexual health education. The 3^rd^ theme of Patient Profile was about the type of patients for which the physicians frequently provide care. Finally, the 4^th^ theme of Background Information collected the socio-demographics data of the physicians. These themes were reviewed and discussed by an internal group of mainstream and Iranian-American physicians including family practice, gynaecologists, internists, and paediatricians, all of whom were involved with delivering sexual health care to patients. These physicians were instrumental in creating both the content and the wording of the survey. For this paper, only questions under theme 1a, *“Obtaining sexual history”*, question number 11, with 12 items (Additional file 1), were used and analysed together with the socio-demographic variables (theme 4).

### Validity and reliability

A panel of five experts with expertise in research and teaching on public health and cultural diversity in health care as well as physicians, all of whom were involved with delivering sexual health care with patients, was invited to validate the questionnaire. Most of the questionnaire items were evaluated by them as appropriate and relevant to the study, with the Content Validity Index equal to 0.90. Minor amendments were made to the wording and order of the questionnaire to achieve a more logical layout [55]. This process resulted in an initial 34-item questionnaire which was then reviewed by an independent group of expert researchers for opinions on the questionnaire’s content validity and face validity. Before the commencement of the study between May and August of 2013, a pilot study was then conducted among 11 physicians to test the comprehensibility of the items and to establish the reliability of the questionnaire. The physicians in the pilot group were selected by contacting a group of researchers that consisted of various types of physicians at Loma Linda University School of Medicine, plus gynaecologists and family physicians in private practice. The overall Cronbach’s alpha of the pilot study was calculated to be 0.9, indicating that the instrument has a high level of internal consistency. The pilot testing resulted in further refinement of the questionnaire.

### Ethical considerations

Ethical approval was obtained from the Human Research Ethics Committee of the University of New England, Australia.

For all the participants recruited, participation was voluntary and no incentive for participation was provided. Physicians’ response to the questionnaire served as their consent to participate as it was described in the cover letter. Questionnaires were anonymous and individual physicians could not be identified by the researchers. The primary researcher is in possession of the returned questionnaires.

### Data collection

The self-administered survey was mailed to the 1,550 selected Iranian-American physicians in California with a cover letter explaining the study and requesting their participation. A self-addressed and self-stamped return envelope was included in the mailing. In order to increase the response rate, a total of three contact attempts were made with the target participants. Four weeks after the initial mailing, all 1,550 physicians received a replacement packet similar to the first one, which included a thank you letter. A week after the second contact, reminder phone calls were made to a random 50 % sample of the 1,550 physicians in our target study population. The timeframe to receive the questionnaire and responses was 30-45 days and data collection took place between May – August of 2013.

### Statistical analysis

The collected data was entered into Excel 2010 and data was analysed using SPSS 22 and SAS 9.4. The statistical analyses included descriptive statistics, exploratory factor analysis, correlation, two sample t-tests, regression and ANOVA. The exploratory factor analysis employed a principal component approach (PCA) with Varimax rotation. All 11 questions in the section “What are the barriers you have experienced when obtaining sexual histories?” (Theme 1a) (Additional file 1) were utilized for this. The exploratory factor analysis identified three meaningful factors based on the eigenvalues (the amount of variance accounted for by each factor) and scree plot test [56]. Items loading at ≥0.5 were retained for factor clarification and conceptual description. This choice in general is subjective, although the guideline was based on literature [57] as follows: range ≥0.30–0.47 was graded as weak, 0.48–0.60 graded as moderate, and >0.60 were strong. We checked the internal consistency via Cronbach’s alpha as a measure of reliability statistics [58]. The Cronbach’s alpha reliability coefficient normally ranges between 0 and 1, and 0.7 is to be an acceptable reliability coefficient [59]. The following rules of thumb applies: “_ > .9 – Excellent, _ > .8 – Good, _ > .7 – Acceptable, _ > .6 – Questionable, _ > .5 – Poor, and_ < .5 – Unacceptable” ([60], p. 231).

## Results

A total of 354 (22.8 %) physicians returned a completed questionnaire. Characteristics of the respondents are provided in Table 1. The majority were male (57.3 %) and more than half of them were younger than 60 years of age. More than 80 % were born in Iran, about 54 % received their education in Iran, and the majority of them (55 %) were Muslims. Factor analysis was conducted with the eleven Likert scale (1-Strongly Disagree, 2 – Disagree, 3 – Neutral, 4 – Agree, 5 – Strongly Agree) responses that pertained to the barriers that Iranian-American physicians experienced when obtaining sexual histories. Considering factors with eigenvalues larger than one and scree plot, three factors with 77 % variability were selected (*physician’s embarrassment, culture and religion, time and financial constraint*). Communalities, which measured the variance in the responses explained by the factors, were calculated, and since none of the questions produced communalities below 0.6, all were included. When high factor loadings (more than 0.50) were present across the three factors (Table 2), the questions were included in the factors with the largest factor loading value. For appropriateness of factor analysis we used the Kaiser-Meyer-Olkin (KMO) measure of sampling adequacy and Bartlett’s test of sphericity. The KMO statistic for this data is 0.80, which is considered very good. Internal consistency was assessed by Cronbach’s alpha. It was 0.91 for factor 1, 0.87 for factor 2, and 0.87 for factor 3. All three were above 0.87 and therefore were at least good [58].

**Table 2:** Factor loading values along with Cronbach’s Alpha

	Factor1	Factor2	Factor3
Factor 1: Embarrassment (α** = 0.91)			
I feel embarrassed with Iranian females	0.76471		
I feel embarrassed with Iranian males	0.77918		
I feel embarrassed with non-Iranian females	0.89086		
I feel embarrassed with non-Iranian males	0.88646		
Factor 2: Cultural and religion (α = 0.87)			
My religion does not allow it		0.81285	
A family member present with the patient		0.50160	
My culture doesn’t allow it		0.85946	
I have not had enough training in obtaining sexual history		0.69278	
Fear of patients taking it personally		0.73668	
Factor 3: Time and financial constraint (α = 0.87)			
Lack of time			0.89156
Lack of reimbursement			0.88676
Values less than 0.5 are not printed.			

Factor Loading below 0.5 are left blanked for clarity of reading. **Cronbach Alpha

Factor 1 included four variables that accounted for 55 % of the explained total variance (eigenvalue of 6.0) from the 11 variables. This factor was composed of *physician’s embarrassment* in obtaining sexual histories from patients: the non-Iranian patients have higher loading factors. The second factor included five variables and accounted for 13.8 % of the explained total variance (eigenvalue of 1.52). This factor, *culture and religion*, prevents obtaining sexual histories with high loading factors on each variable with slightly higher on culture and religion. Factor 3, *time and financial constraint*, consisted of two variables and accounted for 8.5 % of the explained total variance (eigenvalue of 0.93). It included strong loading on lack of time and lack of reimbursement almost equally. From these three factors, approximately 77 % of the explained variance was accounted for.

Significant associations were found between the first factor (physicians’ embarrassment) and gender: Female respondents had higher factor scores than males on the first factor, indicating female physicians experience more embarrassment (Table 3). Marginal significant differences were also found between religion and scores on the first factor. However, no relationship was found between scores on the first factor and country of medical education, type of practice and place of practice. For the second factor (physicians culture and religion), age, place of birth and country of medical education were found to be significantly different. Physicians were influenced by their culture and religion, in particular younger participants (see Table 2). In the third factor (physicians time and financial constraints), the physicians who graduated in Iran experienced greater constraint on time and financial issues. Older physicians experienced more barriers with respect to time and financial issues than younger physicians. It is worth noting that female physicians experienced less constraint on financial issues than did their male counterparts and this was significant (*P* = 0.046). Additionally, when doing simple regression on age as a continuous predictor it was found that age is negatively associated with embarrassment (Factor1), although statistically not significant, but positively and significantly associated with Factor 2 and Factor 3 (*P* < 0.01).

**Table 3:** Comparisons of characteristics (factor scores) for the three factors

	Factor 1		Factor 2		Factor 3	
	Mean (SD)	P*	Mean (SD)	P	Mean (SD)	P
Gender^a^						
Male (*n* = 185)	-0.09 (1.02)	**0.04**	-0.01 (1.01)	0.87	0.09 (1.00)	**0.046**
Female (*n* = 126)	0.14 (0.95)	--	0.01 (0.99)	--	-0.14 (1.00)	--
Place of Birth^a^						
Iran (*n* = 270)	0.00 (0.99)	0.88	0.07 (0.98)	**0.005**	0.03 (1.00)	0.31
Other (*n* = 33)	-0.03 (1.10)	--	-0.46 (1.21)	--	-0.16 (0.92)	--
Religion^b^						
Christian (*n* = 33)	-0.15 (0.89)	0.51	0.11 (1.14)	0.51	-0.36 (1.04)	**0.09**
Jewish (*n* = 69)	0.22 (1.22)	**0.08**	0.16 (1.09)	0.20	0.16 (0.98)	0.16
Other (*n* = 28)	-0.30 (0.83)	0.17	-0.36 (0.74)	**0.09**	0.13 (1.04)	0.43
Muslim (*n* = 179)	-0.03 (0.93)	--	-0.02 (0.96)	--	-0.03 (0.98)	--
Country of Medical Graduation^a^						
Iran (*n* = 172)	-0.00 (0.99)	0.96	0.19 (1.07)	**0.0001**	0.11 (1.05)	**0.02**
USA & Other (*n* = 133)	+0.00 (1.01)	--	-0.25 (0.86)	--	-0.16 (0.91)	--
Clinical Specialty^b^						
OB/GYN (*n* = 46)	-0.07 (0.76)	0.97	-0.08 (1.00)	0.51	-0.10 (1.11)	0.36
Family Prac (*n* = 59)	0.16 (1.19)	0.96	0.17 (1.28)	0.99	0.21 (1.13)	0.98
Urologist (*n* = 26)	0.05 (0.88)	0.97	-0.16 (0.76)	0.89	-0.18 (0.89)	0.86
Other (*n* = 180)	-0.04 (1.00)	--	-0.01 (0.92)	--	-0.02 (0.94)	--
Place of Practice^b^						
Suburban (*n* = 167)	0.05 (1.01)	--	0.05 (0.95)	--	0.07 (0.94)	--
Urban (*n* = 126)	-0.06 (1.00)	0.93	-0.09 (0.99)	0.77	-0.06 (1.06)	0.50
Rural (*n* = 16)	-0.04 (0.94)	0.99	0.23 (1.54)	0.45	-0.23 (1.15)	0.80
Age^b^						
30-39 (*n* = 34)	0.22 (1.14)	--	-0.35 (0.83)	**--**	-0.45 (0.91)	--
40-49 (*n* = 106)	-0.04 (0.97)	0.69	-0.24 (0.78)	0.961	-0.03 (0.96)	0.19
50-59 (*n* = 67)	-0.07 (0.86)	0.64	0.31 (1.14)	**0.011**	0.04 (1.01)	0.13
> = 60 (*n* = 99)	-0.05 (1.04)	0.66	0.201.07)	**0.012**	0.18 (1.03)	**0.02**
Test of Trend						
Age (Trend Test)	-0.00754**	0.17	0.01683	**0.002**	0.01632	**0.003**

^a^ *t*-test is used

^b^One way ANOVA with Tukey multiple comparisons was used

*P is the p-value and it is considered significant at 0.05. ** Slope of age

Bold data are significant p value

As a sensitivity analysis, we itemized patients’ gender vs. physicians’ gender in a sub-analysis to identify possible gender bias among physicians. The result shows that male physicians have slightly higher level of embarrassment towards female patients than with male patients. However, this is not significant. Our findings suggest that there is no significant physician gender disagreement about embarrassment related to female patients in general (*p* = 0.511 for Iranian female patients; *p* = 0.848 for non-Iranian female patients). However for male patients in general, female physicians have higher level of embarrassment than male physicians (*p* = 0.001for Iranian male patient; *p* = .006 for non-Iranian male patients) (Table 4).

**Table 4:** Patient’s gender vs. physician’s gender sub-analysis

		Physician’s Gender		
I feel embarrassed with		Male	Female	P_Value
Iranian females	Disagree	122 (61 %)	85 (65 %)	0.511
	Agree	77 (39 %)	46 (35 %)	
Iranian males	Disagree	**141 (73 %)**	**67 (52 %)**	**0.001**
	Agree	**52 (27 %)**	**62 (48 %)**	
Non-Iranian females	Disagree	141 (71 %)	92 (70 %)	0.848
	Agree	57 (29 %)	39 (30 %)	
Non-Iranian males	Disagree	**148 (76 %)**	**80 (62 %)**	**0.006**
	Agree	**47 (24 %)**	**50 (38 %)**	

Bold data are significant p value

## Discussion

To our knowledge, this pilot study is the first to attempt to assess barriers to providing sexual health care among Iranian-American physicians in California. We acknowledge that our survey response rate was low, however, not inconsistent with other mailed surveys among physicians [61–67]. There could be several reasons for the low response rate including time restraints, lack of interest or sensitivity of the topic. In spite of the low response rate, however, the survey elucidates the challenges that this subpopulation of US physicians may have with respect to providing adequate sexual health care. This is a culture that continues to enforce a taboo against open expression of sexuality. Consequently, this type of taboo may cause Iranian-American physicians to have many concerns about raising the issue of sexually related topics with their patients. Not surprisingly, this may also result in an adverse effect on their willingness to answer sexual health care surveys.

Research suggests that offering an incentive may cause a biased response rate [68]. Studies that have offered both $20 and $50 check incentives to physicians report higher response rates than ours [68, 69], but we consider use of such incentive ethically questionable. In our case, no incentive was offered, and therefore, we consider the responses made to our survey to be less biased although we cannot rule out other types of response bias.

Specific steps that may improve the participation of the Iranian-American physicians may include making the survey available to be completed online and/or doing a media campaign prior to the survey to raise awareness of the importance of this topic. In spite of our low response rate, however, the findings of our study can contribute significant information to the development of educational strategies in sexual health care. While our findings illuminated similarities in the existing barriers among the mainstream physician population and our target sample, as noted earlier in the Introduction [18, 20–24], our analysis justifies the need for more attention to cultural biases that may serve as an additional barrier to proactive communication of sexual health care among foreign-born/subpopulation physicians themselves. Thus far, research has demonstrated the importance of cultural competency when interacting with patients [1, 20]. Equally important is the extent to which a physician’s personal culture may impact his or her communication level in their practice as the communication gaps can hamper effective sexual health care [21]. There remains much to be learned about forming an effective method of communication among patients and physicians with ethnic backgrounds such as the Iranian-American physicians in the United States.

According to our data, culture and religion (Factor 2) served as the prime barrier to proactive communication about sexual health care among this population. Within factor 2, place of birth (*p* = 0.005), country of medical education (*p* = 0.0001), age of physicians (50 and older vs < 50) (*p* = 0.011) were found to be significant barriers. Religion was marginally significant (*p* = 0.09). Barriers further encompassed cultural sensitivity toward patients. The results suggest that a direct exposure to home culture may be a strong cultural factor which serves as a barrier to providing sexual health care to their patients in the US. Our findings suggest that, independent of gender and practice specialty, Iranian-American physicians, as providers of sexual health care, may hold specific cultural biases such as roles and value systems. These biases may potentially function as barriers when communication about patients’ sexual history, needs to be initiated by the Iranian-American physicians. To some degree, this barrier seems to be related to place of medical education, e.g. graduating from Iranian medical schools versus obtaining medical degrees and training in the US. Our findings are supported by a study from Australia showing that the Iranian culture plays a role in female sexual health care delivery among the Iranian-Australian physicians as they primarily focus on reproductive care instead of all other aspects of sexual health care [49].

The clinical approach to sexual health care begins with taking a patient’s sexual history [37, 70], and primary care physicians have reported feeling uncomfortable taking sexual histories from patients [30]. Our data support these findings in that our study suggests that variables such as inadequate training and the physicians’ and patients’ genders serve as barriers that influence the level of communication of the physicians when obtaining sexual histories from their patients.

Our findings that family support (family member presence in the examination room) of a female patient visiting Iranian-American physicians also serves as a barrier to sexual health care (see loading factor 0.502 for family member present in Factor 2, Table 2), is in line with findings from another survey study [68] which showed that the issue of family members’ involvement is a cultural barrier to receiving health care in Iran. This is even more so for sexual health care since a female patient is usually accompanied by another female family member, when visiting physicians. Prior studies suggests that due to limited privacy and lack of confidentiality, Iranian women have learned to keep their sexually related issues secrets [39]. Based on our survey, it seems that many Iranian-American female patients are accompanied by a female relative and thus do not have the same privacy and confidentiality normally found in mainstream patient-doctor interactions.

The aforementioned comments suggest that the issue of patients’ keeping secrets (in particular women) creates specific vulnerability to certain kinds of illnesses since the patient cannot be treated for them if he/she is too embarrassed or fearful to express them to his/her doctor. An investigation [49] about the role that Iranian culture plays in health care supports these findings by suggesting that limited education and training in Iran’s medical schools still impacts sexual health care among these physicians, as they primarily focus on reproductive care instead of all other aspects of sexual health care.

Limited training as a barrier includes unfamiliarity about when, how, or what to ask patients. Barriers further encompass cultural sensitivity toward patients. Cultural sensitivity has also been described as a well-known barrier among mainstream physicians related to the reluctancy to regularly take sexual histories from patients from the Hispanic, Asian, African- American and other cultures [29].

Our survey shows that the time spent with patients plus financial constraints [71] are among the barriers that impact our sample physicians’ proactive communication level as they relate to their interactions and clinical approaches to patients when providing sexual health care services (Factor 3). Literature supports this finding also among the mainstream physicians [72]. Our finding shows that female physicians have lower score on this factor (*p* = 0.046), indicating that they spend more time with patients in regards to sexual health care services. Also, there is a significant difference among physicians depending on where they received their medical training with those who graduated from Iranian medical schools having higher scores, indicating spending less time on sexual health care (*p* = 0.02) compared to US graduates. This is also true for participants older than 60 years of age compared to younger physicians (*p* = 0.02).

Our study also found that there was a significant difference to proactive communication on embarrassment (Factor 1). Males and females had significantly different scores (*p* = 0.04) and borderline significant differences were also observed for religion (*p* = 0.09). The implications of these barriers can potentially result in delayed or incorrect diagnosis and insufficient prediction of the level of risk that patients may experience with respect to diagnosis and treatment for STIs. Embarrassment may further impact assessment, diagnosis and treatment of sexual dysfunctions which may develop as a result of other illnesses such as cardiovascular disease, diabetes and depression [36], and ultimately reflect on the patients’ quality of life.

Even though this study was done among Iranian-American physicians, it is possible that similar barriers exist in other minority physician populations as well as to a lesser degree among mainstream US physicians. As sexual behaviour changes and sexually related diseases increase, it is important to focus on the preparedness of physicians to routinely take sexual medical histories. Medical schools have a responsibility in this respect. However, only by knowing more about possible barriers within the existing physician sub-populations will it be possible to give adequate continuing education to address and overcome such barriers. More effective and comprehensive training about sexual health will go a long way towards helping patients to trust their doctors in order to facilitate improved sexual health care and be more responsive and responsible in discussing sexual matters with them.

### Limitations of the study

This study has limitations that must be taken into consideration when evaluating and interpreting the results. The study population is limited only to California and not the entire United States. The low response rate, as noted earlier, is a limitation as is the possibility that those who responded are not representative of all the 1,550 physicians who were invited. Further, our findings reflect the perceptions of the physicians, which may differ from their actual behaviours in clinics. Equally important, is the understanding of the views of patients which we did not survey in this study.

Because our study was cross-sectional, it cannot assess causal relationships of age vs. culture or religion and financial constraints as barriers. Finally, our survey was based on self-reported responses which could have resulted in report bias. It would have been useful to interview a proportion of the patients of each of these physicians to also assess the patients’ perception of barriers. In spite of these limitations, we believe this pilot study sheds light on issues that exist among Iranian-American physicians and which may also be present in other subpopulations or mainstream groups of US physicians.

## Conclusion

Our survey found that Iranian-American physicians may experience various barriers related to addressing the sexual health care of their patients. Further qualitative and quantitative studies are needed to learn about the barriers experienced amongst the Iranian-American physicians as well as other subgroups of US physicians as it relates to sexual health care delivery. This should be complemented by similar studies among other physician populations with and without minority backgrounds, to determine whether similar barriers to effective sexual health care are present. Furthermore, studies among similar patient subpopulations are needed to assess their perceptions of the sexual health care they receive from mainstream and ethnic minority physicians. This will result in better knowledge about the adequacy of sexual histories and care and the degree to which it varies by ethnicity, religion, and gender of the physician. It is essential that physicians are able to provide a safe and supportive medical consultation environment so that patients feel secure and confident to share their sexually related medical concerns. An awareness of their own values and biases is necessary for physicians to be able to facilitate an emotionally safe environment for their patients. Allowing the necessary time is also important to be able to both focus on and listen to their patients’ all important needs related to their sexual health care concerns.

## Abbreviations

HIV, human immunodeficiency virus; STI, sexually transmitted infection; US, United States

## Additional file

Additional file 1: What are the barriers you have experienced when obtaining sexual histories? (DOC 29 kb)
