# Supplementary material for: Barriers to sexual health care: a survey of Iranian-American physicians in California, USA
Source: BMC Health Serv Res. 2016 Jul 15;16:263. doi: 10.1186/s12913-016-1481-8 (PMC4946137; doi:10.1186/s12913-016-1481-8)
Supplement: Additional file 1: — What are the barriers you have experienced when obtaining sexual histories? (DOC 29 kb) [file 12913_2016_1481_MOESM1_ESM.doc]

Appendix 1

What are the barriers you have experienced when obtaining sexual histories? *(Please check (✓) only one answer for each statement)*

Strongly Strongly

Disagree (SD) = 1 Disagree (D) = 2 Neutral (N) = 3 Agree (A) = 4 Agree (SA) = 5

SD D N A SA

I feel embarrassed with Iranian females. □1 □2 □3 □4 □5

I feel embarrassed with Iranian males. □1 □2 □3 □4 □5

I feel embarrassed with non-Iranian females. □1 □2 □3 □4 □5

I feel embarrassed with non-Iranian males. □1 □2 □3 □4 □5

My religion does not allow it. □1 □2 □3 □4 □5

A family member present with the patient. □1 □2 □3 □4 □5

My culture does not allow it. □1 □2 □3 □4 □5

I have not had enough training in obtaining a sexual □1 □2 □3 □4 □5

history.

Fear of patients taking it personally. □1 □2 □3 □4 □5

Lack of time. □1 □2 □3 □4 □5

Lack of reimbursement. □1 □2 □3 □4 □5
